# More Active Mums in Stirling (MAMMiS): a physical activity intervention for postnatal women. Study protocol for a randomized controlled trial

**DOI:** 10.1186/1745-6215-13-112

**Published:** 2012-07-20

**Authors:** Alyssa S Gilinsky, Adrienne R Hughes, Rhona J McInnes

**Affiliations:** 1 Sport, Health and Exercise Sciences Research Group, School of Sport, University of Stirling, Stirling, Scotland FK9 4LA, UK; 2 Maternal and Child Health Research Group, School of Nursing, Midwifery and Health, University of Stirling, Stirling, Scotland FK9 4LA, UK

**Keywords:** Physical activity, Postnatal, Health behavior change, Accelerometers, Consultations, Pram-walking, Randomized controlled trial

## Abstract

**Background:**

Many postnatal women are insufficiently physically active in the year after childbirth and could benefit from interventions to increase activity levels. However, there is limited information about the efficacy, feasibility and acceptability of motivational and behavioral interventions promoting postnatal physical activity in the UK.

**Methods:**

The MAMMiS study is a randomized, controlled trial, conducted within a large National Health Service (NHS) region in Scotland. Up to 76 postnatal women will be recruited to test the impact of two physical activity consultations and a 10-week group pram-walking program on physical activity behavior change. The intervention uses evidence-based motivational and behavioral techniques and will be systematically evaluated using objective measures (accelerometers) at three months, with a maintenance measure taken at a six-month follow-up. Secondary health and well-being measures and psychological mediators of physical activity change are included.

**Discussion:**

The (MAMMiS study will provide a test of a theoretical and evidence-based physical activity behavior change intervention for postnatal women and provide information to inform future intervention development and testing within this population.

**Trial registration:**

Current Controlled Trials ISRCTN79011784

## Background

During the year following childbirth there are physical and psychological benefits associated with participation in regular physical activity [1,2]. A gradual increase to a physically active lifestyle following the early postnatal period (six weeks after delivery) has been shown to have positive implications for mood, fatigue, cardiovascular fitness and weight management [3-6]. As seen in the general population, physical activity reduces long-term mortality and morbidity risk for a range of health conditions, including coronary heart disease (CHD), Type II diabetes, obesity, cancer and depression [7,8]. Women with young children have increasingly been targeted through physical activity promotion interventions [4,5,9-18]; however, relatively few studies have focused specifically on postnatal women in the year following childbirth, despite these known health and well-being benefits [4,5,12-18]. There is evidence that many women are insufficiently active during the postnatal period [19-24], suggesting postnatal women represent a target population for physical activity promotion interventions.

To date there has been limited research on the determinants of participation in physical activity among postnatal women. Women’s confidence (self-efficacy) that they can be active in the face of barriers (for example, lack of childcare, tiredness) are important; as are enablers to activity, such as “feeling better”, and the ability to commence and sustain self-regulatory effort towards an active lifestyle [24-26]. Research with mothers of young children has also shown a positive effect of self-efficacy and partner support on mediating change in physical activity participation [11]. Commonly, physical activity promotion efforts in postnatal populations have focused on enhancing motivation for activity, problem solving barriers to participation and teaching behavioral strategies associated with commencing and sustaining self-regulatory effort towards an active lifestyle (for example*,* setting goals, planning and self-monitoring activity). Three small-scale studies, conducted in the USA, Canada and Australia, recruited healthy women in the year following childbirth and successfully used such approaches to change participation in physical activity [12,14,17]. One study, which was conducted among women meeting criteria for postnatal depression (PND), found no change in physical activity in a UK setting [16]. Other studies have attempted to overcome common barriers by promoting activity that can be performed without the need for childcare. In this regard, pram-walking interventions have been developed and tested, originally among Australian postnatal women [18,26]. Pram-walking interventions are appealing as they can be adopted in the local community, provide an opportunity for demonstrating appropriate moderate physical activity, allow women to be active with their babies and provide group support for behavior change. Also, time spent walking appears most resistant to decline during the postnatal period [21,24]; therefore, pram-walking may be an appropriate form of physical activity for this group. However, one evaluation study of pram-walking found no evidence of its effectiveness as an isolated intervention when comparing activity levels between pram-walking mothers and mothers taken from a matched control community. There are also potential issues about transferability to the UK-context and limited information on the acceptability of pram-walking among postnatal women [27].

Most postnatal physical activity intervention trials to date have had methodological weaknesses, including insufficient power [17], no control group [12] or matched controls only [18], reliance on self-report measures of physical activity behavior change and only measuring immediate post-intervention effects [12,14,16,17]. With the exception of Cramp and Brawley [25], there is little information regarding whether interventions effectively changed proposed psychological mediators of physical activity behavioral change. Such considerations are crucial for understanding intervention effectiveness (or ineffectiveness) in order to optimize future interventions [28]. In this article, we describe the rationale and methodological design of the More Active MuMs in Stirling (MAMMiS) study: a randomized, controlled trial testing the efficacy of physical activity consultations using motivational and behavioral techniques, alongside group pram-walking, on participation in physical activity in women who have given birth in the last year (postnatal women).

### Aim

The aim of this study was to test the hypothesis that a motivational and behavioral intervention is more effective than a leaflet for promoting participation in physical activity at three months follow-up among insufficiently active postnatal women. Secondary aims are:

1. To test efficacy at six months follow-up (three months post-intervention),

2. Identify the potential impact of the intervention on physical and psychological health and well-being,

3. Examine the utility of psychological mediators for predicting change in physical activity participation,

4. Assess acceptability of the intervention.

## Methods/design

### Study design

**Figure 1:**
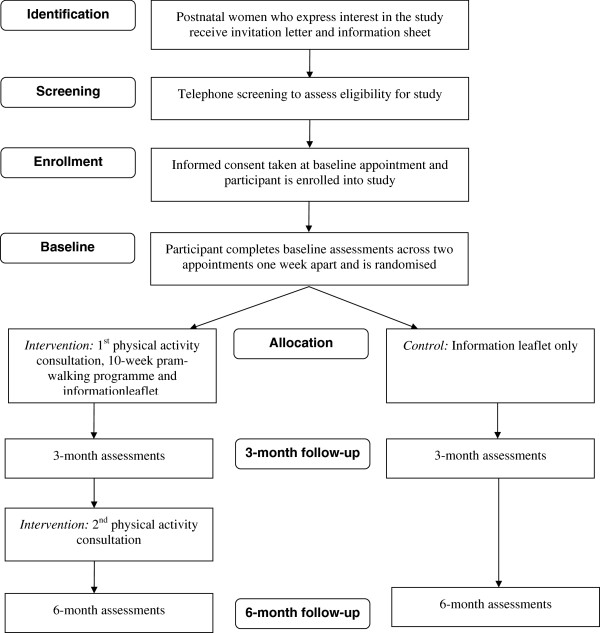
Study Flow.

The MAMMiS study is a single-site randomized, controlled trial (RCT) investigating the efficacy of a motivational and behavioral intervention consisting of two physical activity consultations and a 10-week group pram-walking program on physical activity behavior change. Participants are recruited over a one-year period from a National Health Service (NHS) region within Central Scotland through a variety of strategies (described below). Following eligibility screening, participants give informed consent and complete baseline outcome assessments. These assessments are repeated at three and six months follow-up (Figure 1). The primary outcome is weekly participation in physical activity measured *via* accelerometers. The trial is conducted and reported according to Consolidated Standards of Reporting Trials (CONSORT) guidelines [29] and includes psychological mediators to identify whether change in physical activity occurs through changes to the hypothesized determinants. Acceptability of the intervention is evaluated in a separate study through interviews with participants.

### Recruitment

Participants are recruited from two Community Health Partnership (CHP) areas within a larger NHS region. This region provides primary healthcare services for a population of approximately 185,000 adults and the live birth rate within the targeted region is in line with the Scottish average [30]. The two CHPs targeted cover mixed socio-demographic and geographic areas, including a city, suburban towns and villages and more rural regions. Several different recruitment strategies are used in this study, with advice, support and permission from NHS and local government contacts being used to determine allocation of recruitment resources.

### Recruitment strategies

In Scotland, every woman with a child under five has a named health visitor. Contact with health visitors usually occurs in the early period after birth (for example*,* first at 10 to 14 days), at 6 to 8 weeks postnatal and at 3, 4 and 12 months. In this study, health visitors are provided with advertising materials (study leaflet and posters) to give to potential participants during routine postnatal care. Potential participants are invited to provide contact details to the Chief Investigator (CI) and request further information *via* a postpaid envelope, email or telephone. There is a study website (http://www.mammis.weebly.com) and other strategies include targeting baby and toddler groups, breastfeeding groups, baby reading sessions within local libraries and baby sensory classes (locally-run franchises running private classes for infants and toddlers, available across the recruitment regions). The CI will also conduct on-site recruitment during baby clinics in specific demographic areas. This is used in order to encourage participation from less affluent postnatal women who are traditionally less likely to take part in research studies [31]. Other recruitment strategies include advertisements in local media, participant recommendations and research staff attending local community-based events.

### Eligibility

After women have had the opportunity to review the study information sheet, eligibility is determined through a telephone screening call (Figure 1).

### Inclusion criteria

i) Aged 18 years or older

ii) Have given birth in the last year

iii) Have had a six- to eight-week postnatal check-up with a suitable health professional

iv) Insufficiently active at the level required to promote and maintain health (activity levels are assessed using the Stages of Change questionnaire [32]). Participants are advised that regular physical activity means “accumulating at least 30 minutes of moderate-intensity activity at least five times a week” [33] and are eligible for inclusion if they are in contemplation (not regularly active but thinking about starting to be in the next six months) or preparation stages (participate in some activity but not enough to be considered regularly physically active).

### Exclusion criteria

i) Insufficient English

ii) Pregnancy or those planning to become pregnant in the next six months.

iii) Medical contraindications to physical activity, assessed using the Physical Activity Readiness Questionnaire (PAR-Q) [34].

iv) Stage of physical activity is assessed as precontemplation (not regularly active and no intention to become active in the next six months), action (regularly active in line with guidelines but only began this in the past six months) or maintenance stage (regularly active in line with guidelines and has been so for longer than six months) according to the Stages of Change questionnaire [32]).

No exclusions were made based on characteristics of the baby, that is, women with infants that had been delivered prematurely, had spent time in a special care baby unit and/or had delivered twins or multiple births were eligible for inclusion.

### Sample size

Based on calculations carried out by an independent statistician, 31 participants per group are required in order to detect an effect of 63.83 minutes/week of moderate-vigorous physical activity participation. This assumes power of 90%, 5% significance level with a two-sided unpaired *t*-test to detect change from pre- to post-test between the intervention and control group using a pooled standard deviation of 75 minutes/week. This sample size calculation is based on data from a previous study comparing a 12-week physical activity intervention in insufficiently active breast cancer survivors with usual care [35]. Comparable to the present study, the main outcome in that study was change in weekly minutes of moderate-vigorous intensity physical activity from baseline to three months measured using accelerometers [35]. This study was used as no previous reports of postnatal physical activity promotion interventions had used accelerometers to measure change in physical activity behavior. An increase of 60 minutes of moderate-vigorous physical activity (for example, brisk walking) per week is clinically significant for cardiovascular health [36]. Assuming a 20% dropout (in line with similar studies [14,17]) up to 76 postnatal women will be sought.

### Randomization

Participants are allocated to groups using simple randomization from a computer-generated sequence with block sizes of 4 and 6, generated by an independent person. Following sequence generation, group identifier cards were placed into envelopes alongside a piece of card to block the research team from identifying group allocation. Envelopes were sealed and stacked and participants will be assigned an envelope in the order in which they enroll into the study. This is known as sequentially numbered, opaque, sealed envelopes (SNOSE) [37].

### Intervention

The intervention consists of a face-to-face physical activity consultation (approximately 45 minutes in length) delivered at the start of a 10-week group pram-walking program. Participants receive a second face-to-face consultation (approximately 25 minutes in length) following the three-month assessments. Consultations are structured and individualized counseling sessions that aim to enhance motivation for physical activity and teach participants self-regulatory strategies associated with adoption and maintenance of an active lifestyle [38]. Consultations are structured as they involve a set of standardized techniques but individualized as a discussion focusing on each participant’s personal benefits, barriers, activity goals and plans, and so on*.* Exercise (now described as physical activity) consultations were developed from well-established theoretical models of physical activity change, in particular the Transtheoretical Model (TTM) [38]). Several RCTs have shown individual and group-based consultations to be effective in the promotion and maintenance of physical activity in non-clinical and clinical groups [39-42]. These studies provided two physical activity consultations, 12 weeks apart, alongside support between consultations, often in the form of telephone support. In the present study, the specific motivational and behavioral intervention techniques used have been chosen with reference to research on determinants of postnatal physical activity [24-26] and are drawn from an empirically developed taxonomy of behavior change techniques [43], alongside the many cognitive and behavioral processes of change from the TTM [44] (for example, consciousness raising, stimulus control, and so on*.*)

The consultations will be conducted at the local university or at participants’ homes, depending on preferences, by the CI who has experience and training in behavior change interventions. The first consultation includes the following techniques, introduced to participants through a workbook: awareness raising, information about the benefits of increasing physical activity, specific short- and long-term goal-setting, action planning, self-monitoring through weekly diaries and a pedometer and coping planning. Throughout the process of planning and problem-solving barriers to physical activity, participants will discuss suitable local opportunities for activity and be introduced to the importance of individual-level environmental restructuring (for example, making plans to meet friends on a day when the car is unavailable) and planning social support for behavior change (for example, ask their partner to look after their baby to attend a fitness class). Participants are also invited to attend one group pram-walking session in their local area each week for 10 weeks. A trained walk leader facilitates these and routes have been mapped and risk-assessed as suitable for mothers with prams. Walks are conducted at a moderate-intensity (for example, brisk pace) for 30 to 55 minutes, plus a five-minute warm up and cool down at the start and end of each session. The pram-walking group provides an opportunity to demonstrate a moderate walking pace, provides further social support and prompts for weekly activity. Participants who anticipate difficulties attending are encouraged during the physical activity consultation, to plan alternative physical activity opportunities in line with their personal activity goals. This allows for mothers with difficulties attending walks (for example, due to childcare commitments for older children, transport difficulties or personal preferences). Participants who do not attend the pram-walking group in the first two weeks will receive a 10-minute support phone call encouraging efforts towards being more active and offering an opportunity for problem solving barriers to adopting their activity plan. Following the three-month follow-up period a second consultation will provide personalized feedback on changes in physical activity participation and use relapse prevention strategies to encourage participants to continue with an active lifestyle.

Further details regarding intervention content and approach to development are available elsewhere (A Gilinsky, unpublished submission). Finally, the intervention and control group will both receive a leaflet after baseline assessments. The “Active living during and after pregnancy” leaflet was developed by NHS Health Scotland and provides information on the physical activity guidelines and suitable activities (for example, brisk walking and swimming). The control group will receive no further intervention but will receive standard postnatal management.

### Outcomes

The CI conducts assessments over two appointments during each measurement period (baseline, three and six months), normally a week apart. These take place either at the university or in participants’ homes, depending on participant preference. The usual order of assessments is demographic details, psychological well-being and fatigue, cardiovascular fitness, height (baseline only), weight and body composition, followed by instructions on wearing the accelerometer (appointment one). Participants return their accelerometer at appointment two where they also complete a questionnaire regarding psychological mediators of physical activity and physical activity participation is assessed using the Seven Day Physical Activity Recall (7-Day PAR) interview.

### Physical activity

Physical activity change is measured using Actigraph GT3X and GT3X + accelerometers (Actigraph, Pensacola, Florida, USA) Accelerometers are worn during waking hours for seven consecutive days (excluding bathing and swimming) on the right hip, with movement assessed using sampling intervals (epochs) of 60 seconds. Accelerometers have been shown to be reliable and valid measures of physical activity participation in community samples, including overweight or obese adults [45]. During the measurement week, participants record wearing times using a diary. This aids with identifying non-wear periods; defined in this study as a consecutive string of 0 counts for 45 minutes (drop-time tolerance of three minutes). These criteria were developed following testing with a subset of data generated from study participants and is aligned with criteria used in other studies and best practice guidelines [46-48]. Participants with at least four valid days of data monitoring will be considered as having a valid dataset. A second measurement week is used if wear time criteria has not been met during the first measurement week. In this study, percentage wear-time is used for assessing a valid day. Specifically, a day is considered ‘valid’ if non-missing data are available for at least 70% of common wear-time hours (standardized for the hours each day where at least 70% of participants are found to be wearing the monitor). The benefits of this approach (compared with a more traditional approach including only days with >10 hours data recorded [49]) are that incomplete hours can be counted towards total wear-time. Furthermore, women with infants may keep different waking hours from the general population and may take the accelerometer off and on more frequently (for example, due to sleeping during the day). This approach was used in a recent accelerometry study conducted among pregnant and postnatal women [47].

At the end of the measurement week, the 7-Day PAR interview is administered to participants [50]. This method uses standardized prompts to encourage participants to recall the duration and intensity of activities, such as walking for transport, participation in structured exercise, home and work-based activities, and so on*.* Previous research has found that this is a reliable and valid method for measuring habitual physical activity [51]. The 7-Day PAR provides information regarding activity that is not assessed by the activity monitors (for example*,* swimming). Participants are also asked whether the activity levels during the previous week were “less than”, “more than” or “about the same” as the last three months and provide reasons for deviations from normal activity. This explanatory data will aid in interpretation of trial data.

### Secondary outcomes

#### Psychological well-being and fatigue

Psychological well-being is measured using the Adapted General Well-being Index (AGWI) [52]. The original General Well-being Index was developed in the United States and has been adapted for use in the UK. The AGWI consists of 22 items to assess positive well-being, self-control, anxiety and depression, vitality and general health concerns. Each statement is assessed using a five-point Likert response scale in relation to “the past two weeks” and a total well-being score is created by summing questions (negatively worded questions are reverse coded). The AGWI has been shown to have good reliability and validity with the Montgomery-Asberg Depression Rating Scale [53]. It has also been validated within a GP practice sample in the UK against several relevant criterion measures of subjective well-being, including global health status, reporting of ongoing psychological health problems (for example, depression), contact with health professionals, use of antidepressant medicine, tranquillizers or sleeping pills, common psychosocial worries or difficulties and having been unemployed in the last year [53]. Fatigue is measured using one question through a visual analogue scale (VAS). Visual analogue scales are a commonly used uni-dimensional method of assessing health status [54]. Participants place a mark on a 100 mm line to indicate their fatigue levels where no fatigue = 0 and worst possible fatigue = 100. The response category will be whether or not participants have been “affected by fatigue in the past two weeks”.

#### Cardiovascular fitness

Cardiovascular fitness is measured using the Chester Step Test [55]. The Chester Step Test is a sub-maximal fitness test that involves asking participants to step up and down on and off of a standardized step repeatedly in two-minute slots up to a maximum of ten minutes. Stepping rate is determined by a beep that increases after each slot. Participants wear a heart rate monitor (Polar Wearlink WIND chest transmitter, Polar Electro Inc., Lake Success, NY, USA with readings transmitted to a wrist-watch (Polar RS800, Polar Electro Inc., Lake Success, NY, USA) Heart rate is recorded at the end of each slot and the Rating of Perceived Exertion (RPE) scale [56] is used to indicate participants’ perception of activity intensity. The test continues until the participant reaches a heart rate (HR) that is 80% of their age-predicted maximum HR (220-participants age) or until they report an RPE of 14 or above (corresponding to perceiving the activity to be at least “hard”). Cardiovascular fitness (aerobic capacity) is predicted using a standardized equation using the number of slots completed and heart rate at termination of the test. The Chester Step Test has good validity as a predictor of cardiovascular fitness, measured against a maximal fitness test and it can be used with adults of all ages, including participants who are sedentary and overweight [55]. The test is conducted according to the Chester Step Test manual [57] and by using a step size of (8”/20 cm) as this is recommended for participants aged 40 and under undertaking little regular exercise. Prior to commencing the first test, participants take part in a two-minute familiarization period to avoid them experiencing a learning effect. Standardized encouragement was provided during the test.

#### Weight, BMI and body composition

Height is measured in centimeters (to the nearest cm) using a stadiometer (Leicester Portable Height Measure, Seca GmBH & Co Kg, Hamburg, Germany). Height readings are taken twice and averaged at the first baseline appointment. Weight and body composition (% fat mass) are measured using the Tanita portable bioelectrical impedance monitor (Tanita 300MA Tanita Europe B.V., Amsterdam, The Netherlands) according to the measurement protocol specified in the handbook (Tanita, technical notes). Weight is measured in kilograms (to the nearest 0.1 kg) and BMI computed as the participant’s weight in kilograms divided by their height in meters squared (kg/m^2^).

#### Psychological mediators

Psychological mediators are measured *via* a self-completion questionnaire that has been developed for this study from adapted measures used in previous studies [58-62]. The reliability of all questionnaire items will be tested in the study using Cronbach’s alpha for each proposed mediator, which are measured using 4 to 7-point Likert scales (for example, very unlikely – very likely, completely untrue - completely true *etc.*). Mediators measured in this study are specifically those targeted by the intervention methods (*for example,* outcome expectancies, self-efficacy, intentions to be active, action and coping planning and self-regulatory actions). Self-efficacy (*for example,* “How confident are you that you can be regularly physically active during the next three months…even if I’m tired”, and so on*.*) and outcomes expectancy measures (“If I were regularly physically active during the next three months…I would be healthier” and so on*.*) were sensitive to the most frequent barriers and enablers to activity cited by postnatal women; taken from a survey 667 postnatal women [63]. Intentions are operationalized using one item: “During the next three months do you intend to be regularly physically active?” [58]. Action planning is measured using four items following the stem: “I have made a detailed plan about being regularly physically active during the next three months…” (for example, how/when/where/how often). Coping planning, in contrast, asks participants to rate three items; for example, the extent to which they “have a detailed plan about what to do if things get in the way of them being regularly physically active during the next three months”. Planning measures have been validated in previous studies [61]. Six items are used to measure self-regulatory effort, adapted from the action control questionnaire used by Sniehotta *et al.*[62]. Example statements are: “During the last three months…I have been aware of how much physical activity I should be doing to meet my personal standards. I have made sure to monitor how much physical activity I’ve done and I have tried really hard to be physically active.”

#### Process measures

In line with CONSORT [29], trial process information will be reported to include information on the number of postnatal women who expressed an interest in joining the study, the numbers who were eligible and drop-outs at each trial stage. Demographic details collected from trial participants will be compared with decliners and ineligible women to assess representativeness in terms of their age, infant’s age, physical activity stage of change and deprivation status as measured by the Scottish Index of Multiple Deprivation (SIMD) [64]. To identify whether the intervention is delivered as intended, attendance at pram-walking groups and consultations will be logged and participants’ use of strategies from the consultations (intervention group only) will be assessed at three and six months. Approximately 20% of the consultations will be recorded and reviewed by another researcher to assess consistency and fidelity of the intervention delivery. Additionally, qualitative in-depth interviews will be used to assess the acceptability of the intervention to the target group and assess possible control contamination. A researcher who is not involved in the main trial will conduct interviews and participants will be made aware that their comments will be anonymized to avoid the primary research team identifying them.

#### Blinding

Blinding of the CI and participants is carried out at baseline since outcomes are taken prior to group allocation. At three- and six-month follow-ups, steps have been taken to minimize potential bias from a lack of blinding of the CI. This includes using objective measures to assess physical activity, fitness weight and body composition according to standardized protocols. Other secondary outcomes are assessed through self-administered questionnaires.

#### Data analysis

Actilife 5 will be used to analyze accelerometer data in the following ways: ‘raw’ accelerometry output (that is, counts per minute (cpm) averaged over the measurement period), time (minutes) spent in moderate to vigorous intensity physical activity per week, continuous bouts of at least 10 minutes of moderate-vigorous physical activity (MVPA) and, percentage of time spent in MVPA. The calculation of MVPA is based on accepted cut-points for adult women in free-living conditions [65-67]. However, there is wide variation in the ability of cut-point equations to accurately assess physical activity in different intensity zones. Cpm is, therefore, an important measure as it provides an estimate of change in total physical activity and has been validated in free-living conditions against criterion methods, such as energy expenditure [68].

Trial data will be analyzed according to intention-to-treat principles with all participants included according to initial group allocation. To minimize the number of statistical tests conducted, Analysis of Covariance (ANCOVA) will be used with a repeated measures design and group allocation as a between groups factor. Covariates, which have been shown to influence physical activity participation, will be included (for example, participant’s age, participant’s BMI and physical activity stage of change). Population-specific covariates (for example, infant’s age and number of children, and so on) will also be added to the model. Bonferroni *post-hoc* testing will be used to investigate intervention efficacy (between-groups change to three months) and assess maintenance effects (whether change is sustained to six-month follow-up). Statistical analysis will be conducted on all secondary outcomes, also using this approach.To assess whether change in the main outcome (physical activity participation) occurred as a result of change in the psychological mediators, mediation analysis will be conducted using the Sobel test, which involves testing the null hypothesis that there is no difference between the total and direct effects, that is, the assumption that there are no indirect effects of the psychological mediators on physical activity behavior change [69]. This approach provides greater statistical power than the more traditional Baron and Kenney [70] approach. Given the small sample size present in the current study, this is an important consideration and should ensure Type II error is minimized.

#### Ethical approval

This study received favorable ethical approval from NHS Fife and Forth Valley Research Ethics Committee and from the School of Nursing, Midwifery and Health Research Ethics Committee, University of Stirling.

## Discussion

Results from this study will be available by the end of 2012. The strengths of this study include the use of a randomized, controlled design, objective measures of physical activity change and a three-month post-intervention follow-up point (six months from baseline). Using this approach, the MAMMiS study will address many of the methodological shortcomings of previous trials. Results from this study may also provide valuable information to inform future physical activity promotion studies with postnatal women. In particular, this trial will provide tentative evidence on the efficacy of a motivational and behavioral intervention including group pram-walking in a UK-setting. As the intervention techniques used in this study have been defined according to previously published taxonomies [43,44] (for specific details on intervention techniques please contact A Gilinsky), the intervention will be easily reproducible. The intervention is potentially generalizable as two physical activity consultations and a 10-week group pram-walking program conducted locally, once per week is at a reasonably low intensity and the intervention could be delivered by a suitably trained person. However, as the present study tests the efficacy of the intervention at a single-site (assessments and interventions are carried out by a single investigator), further testing across multiple sites would be required to address intervention effectiveness in other contexts (for example, different populations, delivery by different researchers/practitioners).

Other strengths of this study include: conducting mediation analysis to help identify whether psychological constructs targeted through the intervention were in fact responsible for changes in physical activity behavior; including health and well-being measures enabling information to be gathered on the potential impact of physical activity change, which may aid in sample size calculations for future studies interested in these outcomes; and finally, the inclusion of a qualitative arm at the end of the trial will help assess intervention acceptability among postnatal women. These features, alongside outcome and process data, will help advance our understanding of physical activity behavior change in postnatal populations.

## Conclusions

Physical activity is important for postnatal health and well-being as well as helping to tackle preventable deaths and improve morbidity outcomes and quality of life through preventing or postponing onset of chronic physical and psychological health conditions. Positive physical activity habits adopted during the postnatal period may also go some way to addressing age-related decline in physical activity found among young and middle-aged women [71]. Thus, the findings from the MAMMiS study will be of great interest to policy makers, health professionals and intervention program planners involved in promoting participation in physical activity, particularly among women in the year after childbirth.

### Trial status

This is an active trial. At the date of submission 65 participants were successfully enrolled.

## Abbreviations

7-Day PAR: Seven day physical activity recall; AGWI: Adapted general well-being index; ANCOVA: Analysis of covariance; CI: Chief Investigator; CHD: Coronary heart disease; CHP: Community health partnership; CONSORT: Consolidated standards of reporting trials; HR: Heart rate; MAMMiS: More active mums in Stirling; MVPA: Moderate-vigorous physical activity; NHS: National health service; PAR-Q: Physical activity readiness questionnaire; PND: Postnatal depression; RCT: Randomized controlled trial; RPE: Rating of perceived exertion; SIMD: Scottish index of multiple deprivation; VAS: Visual analogue scale.

## Competing interests

The authors declare no competing interests.

## Authors’ contributions

ASG is a PhD student and CI for the MAMMiS study with responsibility for the development, conduct and reporting of study. ARH and RJMcI are supervisors to ASG. ARH conceived the original idea and secured funding for the original study proposal. All authors read and approved the final version of the manuscript.
